# Occupational Therapy Goal Achievement for People with Stroke: A Retrospective Study

**DOI:** 10.1155/2020/8587908

**Published:** 2020-04-25

**Authors:** Hashem Abu Tariah, Amal Saud Aljehani, Dana Yasser Alenazi, Dona Abdularhman Alturaif, Malak Nabit Alsarhani

**Affiliations:** ^1^Faculty of Applied Medical Sciences/Department of Occupational Therapy, King Saud bin Abdulaziz University for Health Sciences (KSAU-HS), Riyadh, Saudi Arabia; ^2^King Abdullah International Medical Research Center, Riyadh, Saudi Arabia; ^3^College of Applied Health Sciences, Department of Occupational Therapy, The Hashemite University, Zarqa, Jordan

## Abstract

**Methods:**

A cross-sectional retrospective study design was used. People with stroke receiving rehabilitation services at King Abdulaziz Medical City (KAMC) were the target of this study. Data about the extent of achieving occupational therapy goals were collected using the Goal Attainment Scaling (GAS).

**Results:**

Of the 100 medical records of people with stroke included in this study, only 30% of the goals were functional. Only 54% of the goals were achieved. No association was found between the number of achieved goals and variables such as age, gender, type of stroke, and stroke hemisphere/side.

**Conclusion:**

People with stroke included in this study have not shown significant progress toward achieving their therapy goals. As for clinical practice, this study could guide therapists in the future to improve the process of achieving their patients' goals.

## 1. Introduction

The American Stroke Association (ASA) described stroke as a disease that affects the blood supply to the brain due to a blockage by a clot or a hemorrhage, this means that parts of the brain cannot get the oxygen and the nutrients it needs [1]. Stroke is considered the fifth cause of mortality in the United States of America (USA); approximately 130,000 persons a year die as a result of stroke [1]. It is reported that every 40 seconds, someone in the United States will have a stroke [1, 2]. Unfortunately, there are no statistics about the incidence of stroke in Saudi Arabia.

Depending on the extent of the stroke, occupational therapy practitioners in collaboration with the patient can set realistic functional goals and provide a variety of activities to achieve those goals. Occupational therapy interventions incorporate training on a variety of activities, such as activities of daily living (ADLs), instrumental activities of daily living (IADLs), and work, and/or compensating for lost functions by using adaptive equipment [3, 4]. Therapists also use other rehabilitation approaches with people with stroke such as the constraint-induced movement therapy (CIMT), which is an intervention that includes restraining the unaffected upper extremity in the interest of compelling the use of the affected limb, therefore slowly regaining strength and function [5].

The goal setting step is considered a major factor in goal achievement as it has a role in promoting patient motivation toward achieving their goals and patient adherence to treatment, enhancing treatment outcomes, and improving patient satisfaction [6]. Occupational therapists generate therapeutic goals, in collaboration with the patient, that are patient-centered with the main focus on the patient's lifestyle, needs, roles, and priorities [6]. Patients should participate in setting their therapeutic goals and make decisions in collaboration with the therapist. This in return will enhance the adherence and participation of the patients to their therapeutic interventions since they feel that they have some control over their rehabilitation process and recovery [7]. A study conducted by Ponte-Allan and Giles [8] showed that patients who set functional, independence-focused goals achieved a significantly higher functional outcomes (measured by the Functional Independence Measure (FIM) scores) than those who did not set functional, independence-focused goals [8]. Furthermore, patients who did not make a functional, independence-focused goal statement needed a longer period of stay in the rehabilitation unit [8]. Lavelle and Tomlin [9] reported that all of the 85 people with stroke who participated in their study made progress toward at least one of their rehabilitation goals, each of them had a mean of 2.7 goals, and there was no significant difference between goal achievement with age, gender, side, and time since the onset of stroke [9].

Several studies have been conducted regarding goal setting in rehabilitation. It was stressed that goal setting in rehabilitation is critically important to enhance engagement, motivation, and autonomy in the rehabilitation process [10]. Patient-centeredness and collaboration with patients are necessary for effective goal setting [11]. A systematic review study investigating goal setting approaches that are used in practice among people with acquired brain injury revealed that formal and informal goal setting approaches were utilized [12]. From the reviewed studies, 77% of the clinicians adopted the formal goal setting approaches such as the Goal Attainment Scale (GAS) and the Canadian Occupational Performance Measure (COPM) [11].

However, barriers to goal setting from the perspective of the clinicians were related to factors pertinent to the patients' themselves such as age, gender, communication difficulties, and cognitive abilities [10]. Other barriers were related to the differences in the clinicians' and patients' perspectives of goal setting [11]. Patients need to understand the role of the clinicians in planning and delivering rehabilitation to assist in enhancing their engagement in rehabilitation and identifying rehabilitation goals [10]. To facilitate the process of goal setting, it is important to improve patients' and clinicians' knowledge, experience, skills, and engagement with goal setting [11].

Goals help to direct the end results/outcomes of the occupational therapy process [13]. Occupational therapy goals should be functional and measurable [13, 14]. A common way of writing all components of patients' goals is using the SMART acronym that stands for Specific, Measurable, Attainable, Relevant, and Time-related [14]. SMART goals answer all components (who, will do what, where, how, and by when) needed in the goal [14].

A variety of scales and measurement tools are used to measure occupational therapy outcomes such as the London Handicap Scale (LHS), GAS, and COPM among many others [6, 15]. Therapists can use GAS for evaluating or assessing if patients' goals were achieved or not in the rehabilitation course of intervention. GAS is a statistical analysis and an individualized outcome measure that measures the extent of achievement of patients' goals set by practitioners ([16–19]). Reid and Chesson [18] reported that the GAS is being used increasingly in the USA in rehabilitation facilities to measure rehabilitation outcomes [16, 18]. The significant factors that the GAS depends on are the ability of both patients to achieve their goals and clinicians to predict outcomes based on their knowledge and clinical expertise. Therapeutic goals are more likely to be achieved if they are set with the help of the patient and their family before intervention starts [19].

The Kingdom of Saudi Arabia (SA) is located in the Middle East with a population of more than 28 million. Although stroke is one of the common causes of death in SA, there are no studies that highlight the relationship between stroke as the main cause of death and the lifestyle in the region, and there are no statistics about the prevalence of stroke in SA [20].

The aim of this study was to assess the extent that occupational therapy goals were achieved for people with stroke in a rehabilitation center in SA and to categorize the goals according to the aspects of the domain of occupational therapy. Furthermore, we aimed to investigate if there is a relationship between age, gender, side of stroke, and type of stroke with the number of achieved goals. Another aim of the study was to evaluate the quality of goals written by the occupational therapy staff for the targeted population.

## 2. Material and Methods

### 2.1. Setting and Subjects

King Abdulaziz Medical City (KAMC) is in Riyadh/SA [21]. It was established in May 1983 and has more than 1500 beds and provides all types of care, starting from primary health care to tertiary care [21, 22]. In mid-2003, the rehabilitation department was inaugurated. The rehabilitation services at KAMC consist of physiotherapy, occupational therapy, and orthotics/prosthetics. The occupational therapy department contains different specialties such as orthopedics, neurology, pediatrics, intensive care unit (ICU), hand rehabilitation, and burn, scar, and lymphedema management. However, the majority of cases are referred to rehabilitation in general and to occupational therapy in particular for people with stroke [23]. The medical records of the people with stroke who received rehabilitation services in KAMC in Riyadh were the target of this study after considering the following inclusion and exclusion criteria: people with stroke of both genders who received inpatient or outpatient rehabilitation services especially occupational therapy services between the years 2012 and 2017. Patients with recurrent stroke and/or other neurological comorbidities were excluded from this study.

### 2.2. Study Designs and Data Collection

A cross-sectional retrospective study design was used. Data were collected by a chart review of the participants included in the study. Data were collected by the authors of this study over two months (February and March 2019). Demographic data such as age, gender, and side and type of stroke were collected about each patient included in the study. Data about the extent of achieving occupational therapy goals were collected using the goal attainment scaling (GAS). Goals achieved were evaluated using the five levels for the GAS, which are

- +2, “a much better than expected” level
- +1, “a somewhat better than expected” level
- 0, “the expected level of achievement”
-1, “a somewhat less than expected” level
-2, “a much less than expected” level.

In this study, it was decided to consider zero and above as an indication that the goal was achieved, and below zero if the goal was not achieved. Goals were then categorized according to the aspects of the domain of occupational therapy (occupations, client factors, performance skills, performance patterns, and contexts and environments) (AOTA 2014).

The proposal of this study was submitted to King Abdullah International Medical Research Center (KAIMRC) that is affiliated with KAMC. Institutional Review Board (IRB) approval was obtained (SP 17/213/R) prior to data collection.

### 2.3. Data Analysis

The data collected was entered in MS Excel and then transferred to the Statistical Package for the Social Sciences (SPSS) version 22 for analysis. Descriptive statistics such as frequencies and percentages were used for categorical variables, and means and standard deviations were used for continuous variables. Associations between the rate of goal achievement and some variables (age, gender, and type and side of stroke) were examined using the chi-squared test.

## 3. Results

Medical records of people with stroke who met the inclusion criteria were analyzed. The reviewed medical records were 157, but the complete ones (the medical records with complete documentation) included in the study totaled 100. The mean age of the 100 people with stroke was 59 years old, and the age range was between 23 and 87 years old. Males represented 62% of the sample. Most of the people with stroke included in the study (61%) had an ischemic stroke. Left-sided stroke represented 47% of the sample (Table 1).

The total number of goals for all patients was 249, which gives an average of 2.5 goals per patient. The goals were then categorized according to the aspects of the domain of occupational therapy, where most of the goals fell under “performance skills” (52%). Only 2% of the goals fell under “performance patterns,” and another 2% fell under “context and environment” categories. Functional goals, goals that fell under the category of “occupation,” represented only 30% of the goals (Table 2). Goals that were achieved represented 54% of the total number of goals. Goals that were least achieved related to the category of client factors (37%). Occupational/functional goals that were achieved represented 56% of the goals of that category (Table 2). The authors of the study were divided into two groups, and each of the groups categorized the goals separately. A meeting was held afterwards. Both groups were in agreement with the majority of the categorized goals, the variations were discussed, and a unified categorization was generated. The same procedure was followed to obtain the GAS scores.

In comparing variables such as age, gender, type of stroke, and stroke hemisphere/side with the rate of goal achievement, there was no significant difference found among the groups (*P* > 0.05) (Table 3). As for the quality of goal writing, it was noticed that occupational therapists in the designated setting did not follow the standardized occupational therapy goal writing format (SMART goals).

## 4. Discussion and Implications

The purpose of this study was to assess the extent of achieving occupational therapy goals for people with stroke and categorize the goals according to the aspects of the domain of occupational therapy. Not surprisingly, most of the people with stroke in this study were male patients; this is consistent with the incidence of stroke worldwide, and the most frequent type of stroke was the ischemic type.

The average number of goals per patient was similar to that in other studies [9]. However, only 54% of the goals in this study were achieved. Further investigations were done to explore reasons behind low goal achievement. One of the reasons mentioned by the occupational therapists is the short stay of people with stroke in the acute setting, that is, 5-7 days, which makes it impossible to achieve the goals. The lack of control of the occupational therapists over the length of stay and their short notice about patients' discharge were other reasons behind their inability to achieve all goals. This indicates the necessity to collaborate and coordinate the efforts of all health team members especially the physicians and the therapists to make a holistic treatment plan, and once the patient is medically stable, he/she should be transferred to a lower level of medical care with more focus on rehabilitation rather than discharging him/her home. Occupational therapists also need to reevaluate the effectiveness of their interventions as they are linked with the stated patient-centered goals.

Another aim of this study was to evaluate the quality of the goals written by the occupational therapy staff for the participants. Contrary to other studies that stressed on the importance of writing functional goals [3, 4, 6–8, 10–12, 15], only 30% of the goals reviewed in this study were found to be functional goals. Most of the goals (52%) fell under the category of performance skills which include motor skills, process skills, and social interaction skills. This might also explain the low level of goal achievement.

Although there were discrepancies between the number of goals that fell under the categories of occupation and performance skills, the goals that fell under both categories were achieved at the same rate (56%). This might be related to the fact that both categories are interrelated, especially the physical aspect of performing the activity. For example, goals focused on improving grip strength, endurance, manipulation, coordination, and movement that is part of the performance skills category are interrelated with performing various occupations that fall under the category of occupations.

As indicated in other studies, patient-centered functional goals are better achieved than nonfunctional goals [6, 8, 10–12]. In evaluating the reviewed goals against the most common way of writing SMART goals, it was noticed that even the functional goals were lacking more than one component. For example, goals such as “modified independence in showering,” “independent in lower limb dressing,” “independent in grooming activity,” “to be independent in most ADLs (grooming, showering, and UL&LL dressing),” and “to be independent to modified independent in all ADLs (grooming, bathing, toileting, feeding, and dressing)” are missing the time (when the goal will be achieved) and the under what condition or to what extent components (environment, adaptation, position, amount of time to complete the activity, and cueing needed). This is a serious problem that needs to be addressed in all rehabilitation facilities. Occupational therapists as well as other rehabilitation professionals need to be instructed and trained about the value and measurable way of writing patient-centered goals. In-service education and workshops need to be conducted that focus on all steps of the clinical process including functional goal writing. This should be enforced in all rehabilitation facilities. Furthermore, reviewing proper documentation to all patients receiving occupational therapy services should be implemented. Another implication of this study is the need for occupational therapy programs to focus on the occupational therapy process and train students to write measurable patient-centered goals. Clinical supervisors should have a role in reviewing the competency standards of newly hired therapists. Moreover, team work and close interaction between the patient, family, and therapists are necessary in goal setting and achievement. KAMC provides home program services at a limited scale. Home program and environmental modifications, if required, are essential in enhancing goal achievement.

No significant relationship was found between age, gender, type of stroke, and stroke hemisphere/side with the number of achieved goals. This was found to be consistent with other studies [9].

A limitation of this study was including people with stroke regardless of the time since stroke or whether they were inpatients or outpatients. Collecting data from one facility is another limitation for this study that affects its generalizability. Moreover, only the record review method was used. However, this study was the first of its kind in the Middle East region.

## 5. Conclusion

Through retrospective review, the current study demonstrated that 100 patients diagnosed with stroke and were seen in KAMC in Riyadh have achieved 54% of the therapy goals. As for clinical practice, this study could guide therapists in the future to improve the process of achieving their patients' goals. Findings of this study also recommended that therapists should be trained on how to write SMART goals that are functional and patient-centered.

## Figures and Tables

**Table 1:** Demographic data (*n* = 100).

Characteristics	Frequency
Gender	
Male	62
Female	38
Stroke type	
Hemorrhagic	39
Ischemic	61
Side	
Left	47
Right	43
Bilateral	10

**Table 2:** Number of goals for each category of the domain of occupational therapy.

Category	Number (%)	Achieved	Not achieved
		No. (%)	No. (%)
Occupation	75 (30)	42 (56)	33 (44)
Client factors	35 (14)	13 (37)	22 (63)
Performance skills	130 (52)	73 (56)	57 (44)
Performance pattern	5 (2)	4 (80)	1 (20)
Context and environment	4 (2)	2 (50)	2 (50)
Total (%)	249 (100)	134 (54)	115 (46)

**Table 3:** Relationship of various variables and rate of goal achievement.

Variable	Goal achievement			Chi-square	*P* value
	Achieved (%)	Not achieved (%)	Total (%)		
Age (in years)					
<60	56 (41.8)	45 (39.1)	101 (40.6)	0.182	0.670
≥60	78 (58.2)	70 (60.9)	148 (59.4)		
Total	134 (100)	115 (100)	249 (100)		
Gender					
Male	77 (57.5)	69 (60)	146 (58.6)	0.164	0.685
Female	57 (42.5)	46 (40)	103 (41.4)		
Total	134 (100)	115 (100)	249 (100)		
Type of stroke					
Hemorrhagic	47 (35.1)	48 (41.7)	95 (38.2)	1.165	0.280
Ischemic	87 (64.9)	67 (58.3)	154 (61.8)		
Total	134 (100)	115 (100)	249 (100)		
Side					
Right	71 (53)	59 (51.3)	130 (52.2)	0.361	0.835
Left	53 (39.6)	45 (39.1)	98 (39.4)		
Bilateral	10 (7.5)	11 (9.6)	21 (8.4)		
Total	134 (100)	115 (100)	249 (100)		

## Data Availability

The data of this study collected using the Goal Attainment Scaling (GAS) that used to support the findings of this study are included within the article. The GAS data and the demographic information about the 100 stroke survivors used to support the findings of this study are available from the corresponding author upon request.
